# Foreign

**Published:** 1842-02-12

**Authors:** 


### FOREIGN.

*Tenotomy after thirty-four years of muscular contraction.* By G. B. Childs, Esq.

Mrs. M., æt. 36, of short stature, and pitted with the small pox, applied to me on account of a deformity of the left lower extremity, with which she had been affected four-and-thirty years. She stated that, when two years old, she was attacked with small pox, subsequent to which symptoms of weakness in the muscles of the left leg became apparent. For this she underwent a variety of treatment, and deriving no benefit, she was placed under the care of Sir William Blizard, who recommended electricity to be employed daily. This was continued for three months without success. An unequal power of the antagonising muscles now developed itself, and a gradual contraction of the flexor muscles took place. For this no plan of treatment was instituted, and no further notice was taken of the case until she applied to me in December last.

On examining the limb, I found the heel raised full four inches, the natural angle of the tarsal articulation being totally destroyed; and when an attempt was made to place the foot on the ground, the phalanges of the toes alone rested on it; the knee was contracted at about an angle of ninety degrees; the patella perfectly free in its movements; and the leg capable both of flexion and distension within the angle named. She has never suffered from pain or uneasiness about the joint, and when rapid extension was made, it did not convey that sudden jerk to the hand that is felt in bony anchylosis. The thigh was also semi-flexed on the pelvis.

Having made a measurement of the affected limb, from the trochanter major to the external condyle of the femur, and from thence to the external malleolus, it was found to be of the same length as its fellow, and this circumstance, coupled with the absence of all appearance of bony anchylosis, pointed it out as a very eligible case for operation.

Two circumstances alone were opposed to a perfect restoration of the limb, viz. a defective action of the extensor muscles, and the existing contraction about the hip.

The patient being inoculated with the prevailing mania, was desirous, at all hazards, that something should be done to relieve her deformity.

On the second of December last, in the presence of Messrs. Coulson, Gay, and White, I divided the tendo Achillis. The wound having healed, and a sufficient time allowed to elapse, the usual form of extension was employed, and in a month I had the gratification of seeing the foot resume its natural position. By a continued application of friction, a most perfect harmony has been established between the antagonizing muscles, although, as regards bulk, there is a considerable disparity in the two limbs.

From a consultation at the time it was not thought expedient to follow up the operation by a division of the hamstring tendons; a respite was therefore allowed the patient, in order to ascertain the results of the first division.

Three months elapsed before again presenting herself, when she stated that, having already derived much benefit, she was anxious to undergo a second operation. The foot still retained its normal position, and, much to her satisfaction, she was enabled to wear a boot; before her foot was wrapped only in flannel.

On the 26th of February, in the presence of Messrs. White and Gay, I effected a division of the biceps semitendinosus and semimembranosus muscles. The patient being placed in the prone position, Mr. White made extension of the leg, bringing the tendons of these muscles into view. Having passed a small knife first beneath the biceps, then the conjoined tendons of the semitendinosus muscles and semimembranosus muscles, I divided them from within outwards; each separated with an audible snap.

All resistance to extension was now removed, for with a trifling amount of force the leg could be freely extended on the thigh. Nothing was applied to the wounds but simple strapping, together with a roller, passing from the head of the tibia to above the joint. After a few days an apparatus was applied, constructed upon the principles of England's registered splint, and gradual extension made. Under this treatment the limb has become straight, and up to the present moment the powers of flexion and extension remain perfect.*

In consequence of her becoming *enciente*, I was prevented from interfering with the contraction about the hip, which I rather suspect to depend in a great measure on the shortening of the rectus femoris; if such be the case, I should anticipate the most satisfactory results from a division of this muscle, as it appears to me to be the chief agent in opposing a perfect restoration of the limb.

This case, sir, I think interesting, as I am not aware there has been any publicly recorded in which an operation has been performed in one of long standing; it moreover tends to nullify a conviction which might be assumed by a perusal of Mr. Coulson's paper, viz. that in very protracted cases a division of the hamstring tendons would in all probability afford us but a trifling assistance in relieving these deformities, a much more potent obstacle presenting itself in a contraction of the crucial ligaments.

It would be folly to deny the occasional existence of these contractions, the fact is too evident from Mr. Gray's dissection; but I am disposed to regard such an event as dependant only on fortuitous circumstances. If contraction of the crucial ligaments were the necessary consequence of a similar affection of the hamstring tendons, in no case would they be so marked as in those of some years' standing. The recital of the above case has, I trust, sufficiently proved that such is not a general result.

But it may be urged, in favour of this opinion, that contraction of these ligaments might have existed in the case under our present notice, which yielded to subsequent treatment. Had such been the case, the yielding would not have been so tacit, nor do I think extension will prove of any avail when these contractions have taken place. I am therefore disposed to regard Mr. Gray's case as one possessing peculiarities distinct from the general character of these deformities.

As regards the operation, it is simple only when applied to the conjoined tendons forming the inner hamstring. Some caution is requisite before dividing the tendon of the biceps, for should we introduce the knife too high up, great risk will be incurred of cutting through the ischiatic nerve. We must be careful, moreover, to avoid the peroneal nerve, which runs along the inner border of the biceps muscle, and may be easily felt with the finger. This nerve, therefore, should be pressed aside, and the knife made to sweep around the inner edge of the tendon.—*Lond. Med. Gaz.* December 10, 1841.

[It appears very obvious to us that the above case was one in which systematic and gradual extension by means of the an-

* Since the above operation I have attended her during her accouchement. The labour was natural and easy.

gular carved splints and screw should have been faithfully tested before resorting to tenotomy. The whole course of recent experience in this city tends to prove that, in the absence of actual spasm, contracted muscles will rapidly yield, or, as we believe, *grow longer*, under well regulated extension by means which do not prohibit the locomotion of the patient. Mr. Childs' idea that the contraction of ligaments would furnish an insuperable bar to extension is altogether untenable. If old cases of contraction in the knee, now almost daily treated with success, did not directly show its fallacy, the whole history of club-foot, the joints of dancers, and many other deformities, would amply prove the facility with which the ligaments yield to continued extension. Without positively pronouncing the case a specimen of hyper-operation, we must express our deep regret that the knife should ever be held the first resource in cases of this nature.]

---

*Observations on the Nature and Treatment of Various Diseases.*
By Robert J. Graves, M. D.

### REMARKABLE AND UNEXPECTED RECOVERY FROM LARGE ABSCESSES OF THE LUNGS.

(*Concluded.*)

The following cases occurred in the practice of my distinguished colleague Dr. Stokes, who has, with his usual kindness, permitted me to publish them.

Case IV.—Mr. H., a gentleman aged about 22, was attacked with pain in the side, cough, and fever, and, in a short time, with very copious purulent expectoration. Soon after this, the signs of extensive abscess made their appearance in the antero-superior and lateral posterior of the lung. The patient was then considered to labour under tubercular caverns to a great extent.

Shortly after I saw him, when he presented the following symptoms: the whole antero-superior, lateral, and posterior upper part of the left lung sounded extremely dull; perfectly distinct cavernous breathing, with large gurgling and pectoriloquy, were heard from second rib downwards to the mamma, and the same phenomena were audible along the fold of the pectoral muscle, from the axilla to seventh rib. The expectoration was copious, muco-puriform, but not fœtid, and the pulse full, regular, and under 80.

The treatment adopted was palliative; the pulse soon became natural; all hectic fever ceased; the dulness of sound on percussion gradually diminished, and the patient in the course of some months was restored to health, all the signs of caverns having completely disappeared.

Case V.—A child, aged 12 years, was attacked with measles, in the course of which, severe pulmonary symptoms set in; the measles having subsided, the pulse continued quick, skin hot, and breathing hurried; in about ten days, the patient commenced to expectorate a purulent matter of an offensive character. The fœtor of expectoration continued to increase.

I saw the child the third week after the disappearance of measles. The expectoration was copious, of an ash-gray colour, and of a horrible fœtor; in fact, the entire apartment was tainted by the smell; the left lung presented nothing abnormal, nor did the upper lobe of right, but the whole region of the lower lobe gave a perfectly dull sound on percussion; loud gurgling cavernous respiration, almost metallic, with a painfully distinct pectoriloquism.

The patient was ordered a milk diet, tonic medicines, and country air, and recovered perfectly well in the course of a few weeks.

Case VI.—Mr. D., aged about 25, high shouldered, and with a remarkable stoop, was attacked with cough in the autumn of 1839. His pulse became quick; he lost flesh rapidly, and presented the usual constitutional symptoms of phthisis, in an early stage. Within a few weeks of the invasion of the disease, Mr. D. began to expectorate from half an ounce to an ounce daily, of a sanious purulent matter, having the colour of urine, but not offensive. He soon after came to town; the right clavicle was dull on percussion, the vesicular murmur feeble as far as the third rib; above the clavicle most distinct gargouillement existed, and the same could be heard in the acromial region, particularly when he coughed.

Soon after this the pulse became quiet, and the expectoration, though still preserving the above character, diminished in quantity. The patient went to the Cove of Cork, where he remained for the greater part of the winter season. He returned in spring, having become quite fat, and without the slightest symptom or physical sign of any pulmonary disease.

I could add several similar instances of pneumonic abscesses to those already mentioned, but they seem amply sufficient to prove that the disease is of much more frequent occurrence than is supposed, and is more frequently curable than the serious nature of the lesion would lead us to anticipate.

Some may think that the duration and previous history of the disease may serve to distinguish simple from tubercular abscess of the lungs, but a more accurate examination of facts will shew that no reliance is to be placed upon either as a means of diagnosis, for, on the one hand, tubercular abscess sometimes forms in the course of a few weeks from the apparent commencement of phthisis; and on the other, simple pulmonary abscess is often preceded by inflammation of many months' duration, and the origin and progress of the symptoms are, as in Case II., quite identical with those of phthisis.

It was my intention to have added some observations upon several remarkable cases of phthisis which have occurred in my own practice, and the practice of Dr. Stokes, and in which the patients recovered either temporarily or permanently in a manner quite unforeseen and unexpected. In some, recovery took place after the occurrence of abundant tubercular deposition and crepitus, and in others, after the formation of tubercular cavities.

When the disease was produced by the operation of accidental causes in constitutions apparently sound, the recovery was not so surprising; but we have witnessed recovery in many of a phthisical constitution, and several members of whose families had previously fallen victims to consumption.

Facts such as these ought to prevent the practitioner from placing too great reliance upon stethoscopic examinations, as a positive means of prognosis; for it may be looked upon as established, that phthisis, like most other diseases, *does not always necessarily progress to a fatal termination.—Dublin Journal of Medical Science.* Jan. 1842.

[Few medical authors are more popular in America than Dr. Graves, and we are always pleased to meet with his interesting communications in the Dublin Journal. The cases which he has published on cavities in the lungs seem to require some remarks.

We do not agree with him that cavities of the lungs are regarded, per se, as indications of a necessarily fatal form of disease. Whenever they result from acute pneumonia, they *generally* terminate favourably, the morbid action ceases with the formation of pus, and there is no difficulty in the expectoration of it. We have not unfrequently, particularly in the years 1834–5, found undoubted signs of purulent abscesses, with copious expectoration of pure pus, cavernous respiration, and gurgling, and the patients afterwards perfectly recovered. Gangrene of the lungs terminating in abscess not unfrequently get well. The only danger arises from the extension of the disease to a very large portion of the lungs, and the consequent sinking of the patient. There is also a slow variety of inflammation nearly connected with gangrene, and occasionally passing into it, which is not uncommon in the insane, and will, in a certain proportion of cases, though less frequently than in acute inflammation, terminate favourably.

Tubercular disease is on a different footing; the mere existence of a cavity proves little of itself, but as it is known to be generally connected with extensive tuberculous deposits in the pulmonary tissue, it is in this way in most cases a fatal sign. Still, it is not infallible; for the tuberculous deposit may have advanced very far in certain portions of the lungs, but be still very slightly developed in the other. We agree with Dr. Graves, that the general condition of the patient and the constitutional disorder is the best test of the danger of the affection, and, of course, the surest guide for prognosis.]

*The Medical Examiner* is published every Saturday by *J. C. Turnpenny*, N. E. corner of Spruce and Tenth streets. Terms—*three dollars* a year for one copy, or *five dollars* for two copies sent to the same post office, in all cases in advance. Postage the same as on newspapers. Letters to be addressed,—post-paid,—to the Editors of the Medical Examiner, Philadelphia.

*Reynell Coates, M. D.*, Acting Editor.

*J. B. Biddle, M. D.*, and *W. W. Gerhard, M. D.*, Co-editors.

*J. B. Biddle, M. D.*, Proprietor.

